# A Systematic Review of the Causes and Management of Ischaemic Stroke Caused by Nontissue Emboli

**DOI:** 10.1155/2017/7565702

**Published:** 2017-10-16

**Authors:** Ciaran Judge, Sarah Mello, David Bradley, Joseph Harbison

**Affiliations:** ^1^Acute Stroke Service, St. James Hospital Dublin and Trinity College Institute for Neurosciences, University of Dublin, Trinity College Dublin, Dublin 8, Ireland; ^2^Mercer's Institute, St. James's Hospital, Dublin 8, Ireland

## Abstract

**Introduction:**

The inadvertent or purposeful introduction of foreign bodies or substances can lead to cerebral infarction if they embolize to the brain. Individual reports of these events are uncommon but may increase with the increased occurrences of their risk factors, for example, intra-arterial procedures.

**Method:**

We searched EMBASE and MEDLINE for articles on embolic stroke of nontissue origin. 1889 articles were identified and screened and 216 articles were ultimately reviewed in full text and included in qualitative analysis. Articles deemed relevant were assessed by a second reviewer to confirm compatibility with the inclusion criteria. References of included articles were reviewed for relevant publications. We categorized the pathology of the emboli into the following groups: air embolism (141 reports), other arterial gas embolisms (49 reports), missiles and foreign bodies (16 reports), and others, including drug embolism, cotton wool, and vascular sclerosant agents.

**Conclusion:**

Air and gaseous embolism are becoming more common with increased use of interventional medical procedures and increased popularity of sports such as diving. There is increasing evidence for the use of hyperbaric oxygen for such events. Causes of solid emboli are diverse. More commonly reported causes include bullets, missiles, and substances used in medical procedures.

## 1. Introduction

Embolic strokes are most commonly thrombotic in nature; however, they can also arise from other tissue and nontissue sources. Patients with nonthrombotic, embolic stroke (NTES) can present similarly to those with commoner emboli. Knowledge of the pathological processes and presentation of these conditions is important to enable prompt diagnosis and initiation of management. We analysed the available literature on nonthrombotic, embolic stroke of nontissue origin to create a review of the pathophysiology, causes, and treatment options available for these conditions.

## 2. Methods

An initial literature review was performed to identify potential causes of NTES, and then a formal systematic review was conducted. References were identified by searching EMBASE and MEDLINE databases with no restrictions on publication date between January 1956 and May 2015. Only articles written or translated into English were included. Case series and case reports were included if they discussed cases involving primary diagnoses of embolic cerebrovascular stroke or transient ischaemic attacks arising from matter other than thrombus.

Details of the full search strategy are included in Appendix 1 in Supplementary Material available online at https://doi.org/10.1155/2017/7565702. 1889 papers were identified after duplicates were removed, 248 eligible articles were assessed, and 216 were included in the qualitative summary. All search results were screened by analysis of the title and abstracts, and any potentially relevant articles were read in full text to assess suitability. Articles deemed relevant were then independently assessed by a second reviewer in a nonblinded manner to confirm compatibility with the inclusion criteria. Any disagreements were resolved by consensus. Excluded articles were entered along with the included results into the PRISMA Flow Sheet (Appendix 1) to summarise the selection process. We recorded the disease process described, that is, the source of the embolus. We did not seek to account for demographics or other pieces of information. We categorized the pathology according to source of the nonthrombotic emboli. There was limited risk of bias as we were searching case reports and case series. All data accessed for the study has been submitted to the journal either in the article proper or in Appendix.

## 3. Cerebral Air Embolism

### 3.1. Introduction

Cerebral air embolism (CAE) occurs due to emboli originating in the venous or arterial circulation and are most commonly iatrogenic and traumatic or because of pressure-related pathology. The conditions required for air embolism generally involve direct communication between the source of air and the blood combined with a pressure gradient favouring the passage of air into the circulation [1–4].

Venous air which enters the lungs is filtered by the pulmonary vasculature up to a limit of approximately 50 ml [5]. Above this volume, the filtering capacity is overwhelmed, leading to air passing through the capillaries and into the arterial circulation, thereby causing organ ischaemia. Air may be introduced into the arterial circulation via the venous system, by direct introduction (e.g., surgery or trauma), or by paradoxical embolization through a cardiac or vascular defect, for example, a patent foramen ovale or Atrial Septal Defect. Only 2 ml of arterial air is thought to be required to cause fatal cerebral ischaemia [1, 3, 6].

Estimation of the incidence of CAE can be difficult to its varied aetiology. Studies show that the rate of venous air embolism during central venous catheter manipulation is 0.13%, while during neurosurgical procedures the rate is 10–80%. These figures do not account specifically for CAE [1, 7–11]. One hundred and eighty-nine (87.5%) of the cases and series we found documenting nontissue, nonthrombotic, embolic stroke were due to air unrelated to barometric pathology. Of these, 80% were due to iatrogenic causes. The remainder were caused by trauma, deliberate self-harm, and spontaneous pulmonary pathology (Appendix 1).

Trauma and interventions such as manipulation of central venous catheters, neurosurgery, and percutaneous lung biopsy are the main causes of CAE. Rarer causes include endoscopic retrograde cholangiopancreatography (ERCP) [12–14], colonoscopy [15], varicose venous sclerotherapy [16–20], and hydrogen peroxide washout [21]. Noniatrogenic causes include trauma (commonly affecting the chest), deliberate ingestion of hydrogen peroxide (H2O2) [22], and pulmonary pathology such as bullae rupture [23].

**Figure 1:**
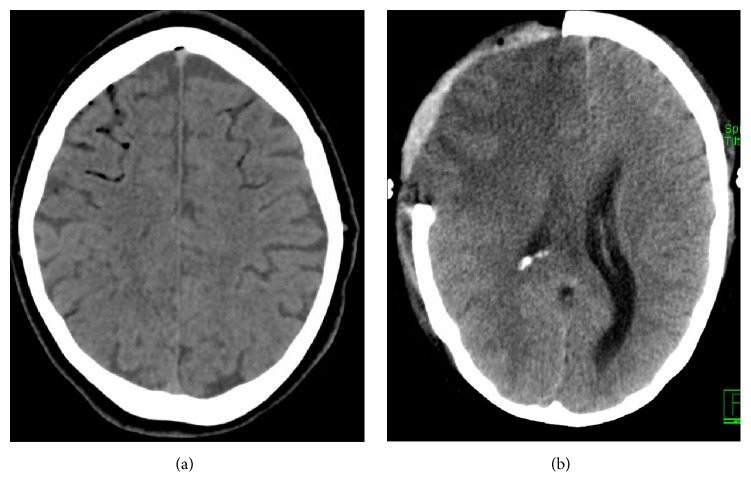
(a) Emergency noncontrast CT scan demonstrating air emboli affecting frontal lobes bilaterally. (b) Repeat CT scan at day four following decompressive hemicraniectomy for consequent infarction.

Cerebral air embolism should be suspected when a patient suffers an acute neurological event following exposure to a risk factor, for example, during percutaneous lung biopsy (Figure 1). The severity depends on the vessels affected and the volume of air introduced. Change in mental status and focal neurological deficits indicate acute cerebral air embolism, and sudden loss of consciousness or coma would suggest a larger volume of air [9, 24]. Investigations may show signs of air emboli affecting other organs (ECG indicating acute MI) or may directly show air within the cerebral vasculature [5, 25].

### 3.2. Treatment

If the patient is suspected as having a cerebral air embolism, the source of the air should be identified and further air entry should be prevented. They should be placed in the supine position. This is due to the inevitable propulsion of bubbles by the force of arterial flow despite being head-down and to avoid any increase in cerebral oedema that may result from being in this position [4]. 100% oxygen therapy should be given which will assist with both respiratory distress and reduction of the size of the bubble. IV fluids should be initiated to increase venous pressure and avoid further entry of gas into the system [3, 26–29].

Hyperbaric oxygen therapy (HBOT), where available, is the recommended treatment for patients with neurological manifestations of CAE. This results in compression of the bubbles, increased diffusion to aid with absorption, improved oxygenation, and reduced intracranial pressure. Prognosis of CAE improves with prompt initiation of HBOT, with best results from early initiation of treatment. However even delayed treatment has been shown to improve outcomes. Transfer should be by ground when possible or at the lowest possible altitude [3, 26–28, 30, 31].

Supportive treatment, inotropes, and admission to ICU are useful in the treatment of patients with CAE. Manual removal of air from the right atrium may be used as a last resort if the patient is in extremis [2, 3, 32]. Use of perfluorocarbons to increase resorption of gas is a potential future treatment; however results have yet to be validated in humans [26].

### 3.3. Prevention

While uncommon, CAE is a potentially devastating condition that is ultimately preventable. Methods to reduce the risk should be employed always. During supine neurosurgical procedures, monitoring of potential venous air emboli with echocardiography in combination with a central venous catheter (CVC) for air aspiration may be utilized [5]. Use of nitrous oxide as an anaesthetic agent can worsen CAE [33]. Patients on mechanical ventilation should have pressures minimized to reduce risk of barotrauma [32]. Manipulation of central venous catheters, a common hospital practice, should be performed in the Trendelenburg position with the patient in exhalation or Valsalva. Other methods to reduce risk include treating hypovolaemia before a procedure or occluding the hub of a CVC during insertion [1, 9, 32, 33].

## 4. Pressure-Related Injury (Barotrauma): Arterial Gas Embolism and Decompression Sickness

### 4.1. Introduction

Air or gas embolism can result from bubbles forming secondary to artificially created pressure-difference arising in various circumstances. These occur more frequently than one might expect. There are perhaps about 1.2 million SCUBA (Self-Contained Underwater Breathing Apparatus) divers in the United States who are at potential risk, and these numbers have been rising over recent decades. The Professional Association of Diving Instructors (PADI) issued over 25 million diver certifications between 1967 and 2016 [34]. Pressure-related injuries can also arise in the aerospace industry in the use of pressurised environments and with developments in medical treatment such as hyperbaric oxygen treatment or even laser ablation of the accessory vein [35–37].

Barotrauma is one of the commonest forms of diving-related injury. The pathophysiology of barotrauma relies on Boyle's law, which states that at a constant temperature the volume of a gas is inversely proportional to the pressure to which it is applied. While descending during a dive, the increasing ambient pressure means the volume of gas-filled spaces, such as the lungs, decreases. If the pressure in these spaces is not equalised by a larger volume of gas, then the space must be filled with tissue, leading to disruption and inflammation. This can result in damage to the affected gas-filled spaces, as well as formation of arterial gas emboli.

Decompression sickness, otherwise known as “the bends” or “Caissons disease,” relies on both Henry's and Dalton's laws, which describes that the inhalation of air under higher pressure when underwater results in the dissolution of increased amounts of oxygen and nitrogen within the tissues [38, 39]. While oxygen is largely metabolised, nitrogen remains inert. If the pressure on the gases decreases too quickly, for example, during a rapid ascent, the gas may expand to the extent that bubbles of free gas are liberated from the tissues. Depending on the volume and location of their release, blockage of vessels, inflammation, tissue disruption, or compression may occur.

Decompression illness is a composite of decompression sickness (DCS) and arterial gas embolism (AGE). Distinguishing between the two conditions is often impossible but may be important in some cases.

### 4.2. Incidence

There are over 1000 injuries and 100 deaths attributable to SCUBA-diving around the world each year. The incidence of DCS is estimated to be 3.6 per 10,000 dives and is estimated to account for almost half of all nondisabling injuries, 1% of disabling injuries, and 2% of deaths [40]. While AGE were less common, they accounted for 9% of disabling injuries and 7% of deaths [40]. Neurological involvement is estimated in 60% of DCS cases, of which cerebral DCS makes up 50–66% [41]. In our review, we found 15 published case series (Table 1) and studies documenting barometrically induced cerebral AGE or DCS. These included cases related to SCUBA-diving, free-diving, an airline mechanic exposed to the decompressed cabin of an aircraft and a woman undergoing hyperbaric oxygen treatment for leg ulcers.

### 4.3. Cause and Presentation

Barotrauma may affect any gas-filled space including the sinuses, middle ear, and lungs. Pulmonary barotrauma is the most severe form but is relatively rare and occurs due to the expanding gases during ascent. Breath holding is the commonest precipitating cause; however underlying lung disease such as asthma can result in air trapping and ensuing barotrauma. Damage to the lung parenchyma can result in entrance of free air into the pulmonary vasculature with the formation of gas bubbles. As these bubbles tend to lodge in vessels 30–60 *μ*m in diameter, ischaemic stroke is a common manifestation [42].

### 4.4. Arterial Gas Embolism

Almost two-thirds of patients with AGE have alterations of consciousness, with seizures, focal deficits, and coma accounting for the majority of presentations [27, 43–45]. Patients with AGE develop symptoms within minutes on surfacing in 80% of cases, or even during ascent. They do not require exposure to significant depth or time underwater to develop AGE. CT or MRI scanning may show focal lesions or the gas emboli within the cerebral vasculature [27, 40, 42].

AGE has significant overlap with DCS and can present simultaneously, making the two conditions indistinguishable in many cases; however differentiation is often made based on suddenness of symptom onset in AGE [40, 46, 47].

### 4.5. Decompression Sickness

Decompression sickness may result from medical-, altitude-, aerospace-, and free-diving-related activities [48]; however SCUBA-diving is the most common cause. Dive profile and ascent time are important risk factors, as are increased age, obesity, dehydration, hypothermia, and the presence of a patent foramen ovale [27, 44, 45, 49].

Focal weakness, stupor, visual loss, gait disturbance, and vertigo are amongst the common manifestations of cerebral DCS [27, 34]. Motor weakness soon after a dive is very suspicious for DCS. It tends to present later than AGE, with 50% developing symptoms within 30–60 mins, and 90% within 6 hours [27, 40, 50]. The spinal cord is the most commonly affected organ which can result in limb weakness, potentially being mistaken for cerebral DCS. Imaging shows lesions that do not enhance with contrast in 30–55% of cases; however many are normal; therefore diagnosis should be made on a clinical basis [43, 46, 51]. Features of decompression sickness are presented in Table 2.

### 4.6. Treatment

Due to the significant amount of overlap between the two conditions, treatment is often identical in the initial stages. Basic and advanced life support, 100% oxygen, rehydration, and Durant's (Left lateral decubitus) and mild Trendelenburg positions should be initiated immediately to restore perfusion to the ischaemic brain. Immediate transfer to the nearest recompression facility for hyperbaric oxygen should be first priority, as early treatment is one of the main determinants of outcome [52–54]. Recompression with hyperbaric oxygen increases the concentration of oxygen supplied to tissues, accelerates the absorption of nitrogen emboli, and reduces the size of bubbles by Boyle's law.

Studies have shown 75% of patients with severe DCS experience full resolution of symptoms if treatment is initiated within 12 hours, which falls to 57% afterwards [54]. This is in contrast to patients with AGE, of whom 50% fully improve with hyperbaric oxygen treatment [55]. 88% of the patients in our review underwent recompression with hyperbaric oxygen. 37.5% of these had a full recovery, 25% died, and the remaining 44% made partial recovery at the time of writing. Patients with residual deficits are recommended to receive repetitive treatment over several days to maximise their chances of improvement [36, 55]. Adjunctive treatments such as steroids and prostacyclin are not currently part of the treatment protocol due to lack of evidence [56, 57].

Urgent transfer to a centre for treatment with hyperbaric oxygen is central to the treatment of both decompression sickness and arterial gas embolism.

## 5. Missiles and Foreign Bodies

### 5.1. Background

Embolization of foreign bodies is a rare and often catastrophic occurrence. There has been a wide variety of materials reported as causing embolic phenomena, ranging from illicit drugs [58] and shotgun pellets [59–65]. Missile embolization is a recognised component of military trauma [64]; however it has also been reported amongst the civilian population [66]. Lower velocity, smaller projectiles used outside of military exercises may actually result in the risk of missile emboli being slightly higher amongst civilians [66].

Illicit drugs are frequently sold diluted and mixed with potentially unknown and hazardous substances [58]. Some Intravenous Drug users inject chemicals not intended for dilution. These vary from hypnotic medications to opiates. If the substance itself or the ingredients it is mixed with are not completely dissolved, then there is a risk of embolization. Cerebral involvement may arise from advertent arterial injection or the presence of a cardiac defect including patent foramen ovale (PFO).

### 5.2. Incidence

**Figure 2:**
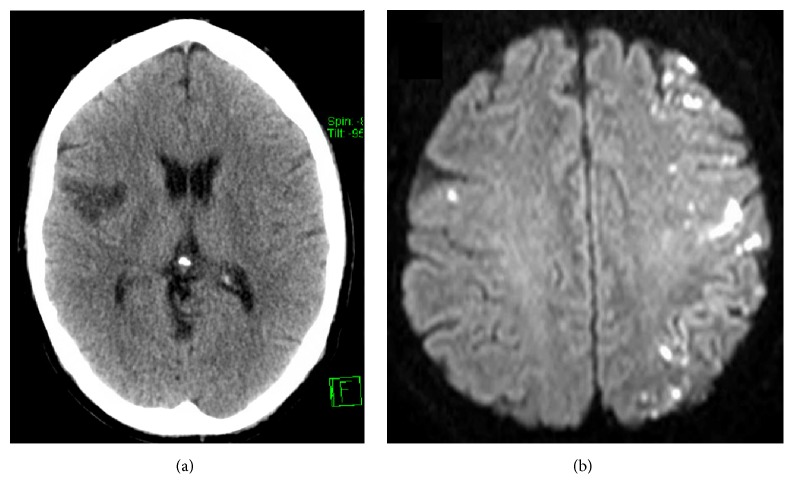
Cerebral infarction in injecting drug users. (a) CT scan showing infarct resulting from paradoxical embolus of injected Zopiclone. (b) MR DWI image of multiple emboli caused by inadvertent injection of diamorphine (Heroin) into left common carotid artery.

Missile emboli resulting from shotgun pellets, bullets, incendiary shrapnel, and other sources are rare. Military studies have shown that the rate of embolic phenomena from missile-induced vascular injury is 0.3–1.1%; however these do not account for cerebral emboli [67, 68]. Studies show that intracerebral emboli have a mortality rate of 25–33% [69, 70]. We found 9 cases describing cerebral missile emboli. Cerebral embolization of illicit material is extremely rare, and we found only 2 documented cases in one report. One involved injection of a partially dissolved hypnotic which embolized via a cardiac defect and the other involved the injection of a mixed opiate into the carotid artery, causing seizure and left hemiparesis [71] (Figure 2).

This is also potentially the case for medications administered through central venous lines. One case of a stroke occurring following administration of cancer chemotherapy through a port-catheter terminating in the right atrium was identified [72] in the presence of a PFO. This event occurred after the second cycle of a carboplatin containing chemotherapy regimen which is a known prothrombotic agent and thus may have been due to a secondary thrombosis rather than the agent itself [73].

Spinal cord infarction following Cervical Transforaminal Epidural Steroid Injection is much more frequently reported, either by secondary thrombosis or by accidental extravasation of particulates within the medications [74, 75]. Such injections have also been reported as a cause of cerebral infarction but mechanism is unclear.

Angiographic intervention commonly results in foreign body embolization, with one study stating up to 25% of resected cerebral AV malformations showing evidence of embolized foreign material after neurovascular intervention [76]. We found 7 series and cases documenting iatrogenic causes for cerebral emboli, with materials including glue, autologous fat, cotton wool, and hydrophilic polymers. The commonest source is related to embolization of cerebral or carotid aneurysms.

### 5.3. Presentation

Focal neurological deficits, sudden disturbance of consciousness, or coma in the appropriate setting should raise suspicion of cerebral foreign body embolus. History of penetrating missile trauma, incongruence of entry and exit wounds, and symptoms out of keeping with the suspected location of the missile could all suggest missile embolus. IVDUs who develop neurological complications following injection or those that inject near arterial sites, such as the neck or groin, should be investigated for potential stroke caused by embolic debris. Patients undergoing procedures, particularly neurointerventional in nature, should be monitored closely for potential embolization of particulate matter. Simultaneous echocardiography or Doppler US of the carotids can be used to screen for such material, and new neurological deficits during or after the procedure would suggest occurrence of an embolic event.

### 5.4. Treatment

Removal of a foreign body depends on presence of symptoms, size, location, and risk of complications. Arterial bullets are symptomatic in 80% of cases while those in the venous system are symptomatic in 1/3 of the time. Complications of arterial bullets include distal ischaemia, thrombosis, and further embolism but may also cause extensive psychological disturbance [77]. Due to the high rate of mortality associated with cerebral foreign bodies, removal is recommended.

## 6. Discussion

Nonthrombotic, embolic stroke (NTES) is often overlooked due to its rarity and the variety of clinical presentation, which contributes to difficulties in diagnosis and management. Treatment and outcomes are often based on individual case reports and case series, meaning that there is a lack of a uniform approach to these patients. Collation of the available data and creation of a stroke register may enable the development of treatment strategies for these rarer forms of stroke.

Nonthrombotic causes of embolic stroke are relatively rare and certain pathological processes are likely to remain uncommon, that is, missile embolism. However, factors such as the increasing availability of interventional and intravascular procedures may lead to a rise in the incidence of other types of NTES. CT-guided biopsies, AV node ablations, and central venous catheter insertions are all associated with cerebral air embolism. There is increasing awareness of the role right left shunts, particularly atrial septal defects and PFOs, which can be a risk in transmitting thrombi and other materials from the venous to arterial circulation, thus risking paradoxical stroke. While reports of this occurring are surprisingly infrequent, this may be because of the difficulties in absolutely proving that the cause of the cerebral artery occlusion was in fact a foreign body or substance without being able to obtain a sample of same from cerebral vessels. PFOs may affect 1 in 4 individuals and establishing cause and effect beyond doubt is difficult in such circumstances but this may change with increased use of neurointerventional techniques in treating stroke. While PFO closure may be of benefit to some patients with recurrent stroke with no other cause found, current guidelines do not currently recommend routine closure [78].

Knowledge of the potential for cerebral events during and after these procedures is essential for early diagnosis and initiation of prompt management. Pre- and postprocedure neurological checklists may flag high-risk procedures and identify early those effected or at risk of cerebral complications. As the neurological consequences of cerebral arterial occlusion by foreign body or substance may differ little from those resultant from thrombotic occlusion, physicians need to be conscious of the circumstances and timing of when the event occurred and, in the era of potential neurointervention, be aware of potential therapeutic interventions.

Due to the small amount of available data, management of these patients is infrequently uniform in nature. Prompt initiation of basic resuscitation and removal of the pathological cause is recommended. HBOT is now thought to be effective in cases of air embolism and decompression sickness and clinicians need to be aware of the symptoms and management of same (Table 2). Embolectomy has been shown to be successful in cases of cervical and proximal cerebral artery occlusion and indeed embolectomy has been demonstrated to improves outcomes in proximal arterial occlusion up to 12 hours from time of onset, in certain cases of thrombotic stroke [79, 80]. This approach may be extended to the mechanical retrieval of foreign bodies and missiles; however, data is scarce. HBOT is a resource which is not readily available in many locations; however with the risk of iatrogenic air embolism and the potential for improvement with treatment, consideration must be given to expand the availability of this therapy [3, 26–28, 30, 31].

Nonthrombotic, embolic strokes are uncommon causes of cerebral stroke and can be easily overlooked. Knowledge of the pathological processes and presentation is essential to ensure that diagnoses are not missed. In the absence of a substantial evidence base there is still validity in the submission and publication of short series and case reports for such events and in particular where anecdotally there may be a risk, for example, medical device failure and disintegration, but there are no published reports of management or outcome.

## Supplementary Material

Appendix 1: List of screened articles by date published and PRISM flow sheet.

## Figures and Tables

**Table 1:** Frequency of reports of decompression sickness and arterial gas embolism by etiology.

Specific causes of DCS/AGE	Number of cases
SCUBA-diving	12
Free-diving	1
Aeronautical industry	1
Hyperbaric treatment	1

**Table 2:** Features of decompression sickness and associated neurological deficits.

Types of DCS	Symptoms and signs	Speed of onset
Type 1	Musculoskeletal pain and cutaneous symptoms such as “marbling” or cutis marmorata	Begin quickly and build in intensity

Type 2	More serious affecting 1 or more of the following	Immediate to delayed
	(i) Neurological: paraesthesia, ataxia, focal deficits, altered mental status. Spinal cord is the commonest site of involvement	Delayed presentation can result in more serious injury due to insidious onset and delay in reporting
	(ii) Inner-ear: tinnitus, vertigo, ataxia, deafness	
	(iii) Cardiopulmonary: dyspnoea, pain, “gasp” due to sudden hypoxia from development of bubbles, cardiopulmonary collapse	
