# Supplementary material for: A Systematic Review of the Causes and Management of Ischaemic Stroke Caused by Nontissue Emboli
Source: Stroke Res Treat. 2017 Oct 16;2017:7565702. doi: 10.1155/2017/7565702 (PMC5662829; doi:10.1155/2017/7565702)
Supplement: Supplementary file 1 — Appendix 1: List of screened articles by date published and PRISM flow sheet. [file 7565702.f1.docx]

Appendix 1

List of screened articles by date published.

1. Sethi JM, Rozdilsky B. Internal carotid artery embolism by shotgun pellet. The Canadian journal of neurological sciences Le journal canadien des sciences neurologiques. 1978;5(3):325-6.
2. Mattox KL, Beall AC, Jr., Ennix CL, DeBakey ME. Intravascular migratory bullets. Am J Surg. 1979;137(2):192-5.
3. Kase CS, White RL, Vinson TL, Eichelberger RP. Shotgun pellet embolus to the middle cerebral artery. Neurology. 1981;31(4):458-61.
4. Marquez J, Sladen A, Gendell H, Boehnke M, Mendelow H. Paradoxical cerebral air embolism without an intracardiac septal defect. Case report. J Neurosurg. 1981;55(6):997-1000.
5. Moser M. [Microembolisms in the capsula interna following decompression accident during diving. A case report (author's transl)]. Laryngologie, Rhinologie, Otologie. 1981;60(7):383-4.
6. Caplan LR, Thomas C, Banks G. Central nervous system complications of addiction to "T's and Blues". Neurology. 1982;32(6):623-8.
7. Bojar RM, Najafi H, DeLaria GA, Serry C, Goldin MD. Neurological complications of coronary revascularization. The Annals of thoracic surgery. 1983;36(4):427-32.
8. Chapman AJ, McClain J. Wandering missiles: autopsy study. The Journal of trauma. 1984;24(7):634-7.
9. Murphy BP, Harford FJ, Cramer FS. Cerebral air embolism resulting from invasive medical procedures. Treatment with hyberbaric oxygen. Annals of Surgery. 1985;201(2):242-5.
10. Bahnini A, Petitjean C, Kieffer E. Gunshot pellet embolus to the middle cerebral artery. Ann Vasc Surg. 1986;1(1):139-42.
11. Aberle DR, Gamsu G, Golden JA. Fatal systemic arterial air embolism following lung needle aspiration. Radiology. 1987;165(2):351-3.
12. Hirabuki N, Miura T, Mitomo M, Kozuka T, Kitatani T, Terashima T, et al. Changes of cerebral air embolism shown by computed tomography. British Journal of Radiology. 1988;61(723):252-5.
13. Warren Jr LP, Djang WT, Moon RE, Camporesi EM, Sallee DS, Anthony DC, et al. Neuroimaging of scuba diving injuries to the CNS. American Journal of Roentgenology. 1988;151(5):1003-8.
14. Marini JJ, Culver BH. Systemic gas embolism complicating mechanical ventilation in the adult respiratory distress syndrome. Ann Intern Med. 1989;110(9):699-703.
15. Kerbler S, Schober PH, Steiner H. [Traumatic embolization of the middle cerebral artery]. Zeitschrift fur Kinderchirurgie : organ der Deutschen, der Schweizerischen und der Osterreichischen Gesellschaft fur Kinderchirurgie = Surgery in infancy and childhood. 1990;45(5):301-3.
16. Uterga JM, Larracoechea J, Fernandez-Rodriguez M, Antiguedad A, Forcadas I, Zarranz JJ. Idiopatic cerebral air embolism. Neurologia. 1990;5(8):288-91.
17. Wolf HK, Moon RE, Mitchell PR, Burger PC. Barotrauma and air embolism in hyperbaric oxygen therapy. Am J Forensic Med Pathol. 1990;11(2):149-53.
18. Black M, Calvin J, Chan KL, Walley VM. Paradoxic air embolism in the absence of an intracardiac defect. Chest. 1991;99(3):754-5.
19. Anda T, Suyama K, Kawano T, Mori K. [Shotgun pellet embolus in the cerebral circulation via the internal carotid artery in the neck; a case report]. No shinkei geka Neurological surgery. 1992;20(4):457-61.
20. Haselsberger K, Oberbauer RW, Piepgras DG. Extracranial-intracranial arterial bypass after cerebral foreign body embolization: Effective treatment of transient ischemic attacks. Neurosurgery. 1992;31(1):141-4.
21. Matsuno A, Hashizume K, Kazekawa K, Suzuki K. [Unexpected air embolism during an aneurysmal operation in supine position--a case report and a speculation about its pathogenesis]. No to shinkei = Brain and nerve. 1992;44(1):77-80.
22. Siccardi D, Primavera A, Tortori-Donati P. Metallic fragment embolization to the middle cerebral artery. Neurochirurgia. 1992;35(4):117-20.
23. Dada MA, Loftus IA, Rutherfoord GS. Shotgun pellet embolism to the brain. Am J Forensic Med Pathol. 1993;14(1):58-60.
24. Egido JA, Arroyo R, Marcos A, Jimenez-Alfaro I. Middle cerebral artery embolism and unilateral visual loss after autologous fat injection into the glabellar area [3]. Stroke. 1993;24(4):615-6.
25. Kol S, Ammar R, Weisz G, Melamed Y. Hyperbaric oxygenation for arterial air embolism during cardiopulmonary bypass. Annals of Thoracic Surgery. 1993;55(2):401-3.
26. Pereira P. A fatal case of cerebral artery gas embolism following fine needle biopsy of the lung. The Medical journal of Australia. 1993;159(11-12):755-7.
27. Sherman SJ, Boyer LV, Sibley WA. Cerebral infarction immediately after ingestion of hydrogen peroxide solution. Stroke. 1994;25(5):1065-7.
28. Carrel T, Maurer M, Tkebuchava T, Niederhauser U, Schneider J, Turina MI. Embolization of biologic glue during repair of aortic dissection. The Annals of thoracic surgery. 1995;60(4):1118-20.
29. Ng WF, Fung KH, Sham JST. Tension pneumocephalus - A rare complication of radiotherapy in nasopharyngeal carcinoma. Pathology. 1995;27(2):204-8.
30. Borgatti R, Tettamanti A, Piccinelli P. Brain injury in a healthy child one year after periureteral injection of Teflon. Pediatrics. 1996;98(2 Pt 1):290-1.
31. Tommasino C, Rizzardi R, Beretta L, Venturino M, Piccoli S. Cerebral ischemia after venous air embolism in the absence of intracardiac defects. J Neurosurg Anesthesiol. 1996;8(1):30-4.
32. Ijichi T, Itoh T, Sakai R, Nakaji K, Miyauchi T, Takahashi R, et al. Multiple brain gas embolism after ingestion of concentrated hydrogen peroxide. Neurology. 1997;48(1):277-9.
33. Reuter M, Tetzlaff K, Hutzelmann A, Fritsch G, Steffens JC, Bettinghausen E, et al. MR imaging of the central nervous system in diving-related decompression illness. Acta Radiol. 1997;38(6):940-4.
34. Schlotterbeck K, Tanzer H, Alber G, Müller P. Cerebral air embolism after central venous catheter application. Anasthesiologie Intensivmedizin Notfallmedizin Schmerztherapie. 1997;32(7):458-62.
35. Mullins ME, Beltran JT. Acute cerebral gas embolism from hydrogen peroxide ingestion successfully treated with hyperbaric oxygen. Journal of toxicology Clinical toxicology. 1998;36(3):253-6.
36. Delgado Reyes S, García De La Rocha ML, Fernández-Armayor Ajo V, Sierra Sierra I, Martín Araguz A, Moreno Martínez JM. Cerebral ischemia in Rendu-Osler-Weber disease. Neurologia. 2000;15(2):76-80.
37. Mitchell SJ, Benson M, Vadlamudi L, Miller P. Cerebral arterial gas embolism by helium: An unusual case successfully treated with hyperbaric oxygen and lidocaine. Annals of Emergency Medicine. 2000;35(3):300-3.
38. Takizawa S, Tokuoka K, Ohnuki Y, Akiyama K, Kobayashi N, Shinohara Y. Chronological changes in cerebral air embolism that occurred during continuous drainage of infected lung bullae. Cerebrovascular diseases (Basel, Switzerland). 2000;10(5):409-12.
39. Yaari R, Ahmadi J, Chang GY. NeuroImages. Cerebral shotgun pellet embolism. Neurology. 2000;54(7):1487.
40. Jahangiri M, Rayner A, Keogh B, Lincoln C. Cerebrovascular accident after vacuum-assisted venous drainage in a Fontan patient: a cautionary tale. The Annals of thoracic surgery. 2001;72(5):1727-8.
41. Soderman M, Bystam J. Cerebral air emboli from angiography in a patient with stroke. A case report. Acta Radiol. 2001;42(2):140-3.
42. Wang HK, Teng MMH, Lirng JF, Luo CB, Chang FC, Chang CY, et al. Iatrogenic cerebral arterial air embolism during cerebral angiogram - A case report. Asian Oceanian Journal of Radiology. 2001;6(2):89-93.
43. Brownlow HA, Edibam C. Systemic air embolism after intercostal chest drain insertion and positive pressure ventilation in chest trauma. Anaesth Intensive Care. 2002;30(5):660-4.
44. Buttinelli C, Beccia M, Argentino C. Stroke in a scuba diver with patent foramen ovale. European Journal of Neurology. 2002;9(1):89-91.
45. Ackerstaff RGA. Transcranial Doppler Monitoring in Angioplasty and Stenting of the Carotid Bifurcation. Journal of Endovascular Therapy. 2003;10(4):702-10.
46. Gomes WJ, Strisiver DA, Penco AJ, Rampersad K, Angelini GD. Successful treatment of accidental air embolism in warm heart surgery. Asian cardiovascular & thoracic annals. 2003;11(1):68-9.
47. Hickey MJ, Zanetti CL. Delayed-onset cerebral arterial gas embolism in a commercial airline mechanic. Aviation Space and Environmental Medicine. 2003;74(9):977-80.
48. Hodics T, Linfante I. Cerebral air embolism. Neurology. 2003;60(1):112.
49. Ackerstaff RG, Vos JA. TCD-detected cerebral embolism in carotid endarterectomy versus angioplasty and stenting of the carotid bifurcation. Acta Chir Belg. 2004;104(1):55-9.
50. Nayagam J, Ho KM, Liang J. Fatal systemic air embolism during endoscopic retrograde cholangio-pancreatography. Anaesthesia and Intensive Care. 2004;32(2):260-4.
51. Parikh S, Narayanan V. Misplaced peripherally inserted central catheter: an unusual cause of stroke. Pediatric neurology. 2004;30(3):210-2.
52. Soto-Sarrión C, Poyatos Ruipérez C, Isarria-Vidal S, Faus-Cerdá R, Esteban-Hernández JM. Arterial gas embolism progressing to a massive stroke. Revista de Neurologia. 2004;38(3):234-8.
53. Ackerstaff RG, Suttorp MJ, van den Berg JC, Overtoom TT, Vos JA, Bal ET, et al. Prediction of early cerebral outcome by transcranial Doppler monitoring in carotid bifurcation angioplasty and stenting. J Vasc Surg. 2005;41(4):618-24.
54. Boghossian T, Henri M, Dubé S, Bendavid Y, Morin M. Laparoscopic nephrectomy donor death due to cerebral gas embolism in a specialized transplant center: Risk zero does not exist [11]. Transplantation. 2005;79(2):258-9.
55. Chessa M, Clai F, Vigna C, Butera G, Negura DG, Giamberti A, et al. Patent foramen ovale in scuba divers. A report of two cases and a brief review of the literature. Ital Heart J. 2005;6(1):73-6.
56. Mellado T P, Constanzo P F, Miquel P JF, Ibáñez L P. Ischemic brain infarction after an air embolism. Case report. Revista Medica de Chile. 2005;133(4):453-6.
57. Roche-Campo F, Mozota-Duarte J, Trigeros-Martín JL, López-Jurado I, Freile-García E, Pina-Leita I. Cerebral gas embolism as a result of diving accident. Medicina Intensiva. 2005;29(4):240-3.
58. Yang CW, Yang BP. Massive cerebral arterial air embolism following arterial catheterization. Neuroradiology. 2005;47(12):892-4.
59. da Costa LB, Wallace MC, Montanera W. Shotgun pellet embolization to the posterior cerebral circulation. AJNR Am J Neuroradiol. 2006;27(2):261-3.
60. Desachy A, Gissot V. Gas embolism during protective ventilation for acute respiratory distress syndrome. Annales Francaises d'Anesthesie et de Reanimation. 2006;25(3):299-301.
61. Forlee MV, Grouden M, Moore DJ, Shanik G. Stroke after varicose vein foam injection sclerotherapy. J Vasc Surg. 2006;43(1):162-4.
62. Rabe C, Balta Z, Wüllner U, Heller J, Hammerstingl C, Tiemman K, et al. Biliary metal stents and air embolism: A note of caution. Endoscopy. 2006;38(6):648-50.
63. Shannon P, Billbao JM, Marotta T, Terbrugge K. Inadvertent foreign body embolization in diagnostic and therapeutic cerebral angiography. AJNR Am J Neuroradiol. 2006;27(2):278-82.
64. Timpert KI, Schmutz J, Steinke K. [Massive cerebral air embolism after computerized tomography guided lung biopsy]. RoFo : Fortschritte auf dem Gebiete der Rontgenstrahlen und der Nuklearmedizin. 2006;178(4):441-3.
65. Tomiyama N, Yasuhara Y, Nakajima Y, Adachi S, Arai Y, Kusumoto M, et al. CT-guided needle biopsy of lung lesions: a survey of severe complication based on 9783 biopsies in Japan. Eur J Radiol. 2006;59(1):60-4.
66. Yamashita Y, Mukaida H, Hirabayashi N, Takiyama W. Cerebral air embolism after intrathoracic anti-cancer drug administration. The Annals of thoracic surgery. 2006;82(3):1121-3.
67. Aghasadeghi K, Aslani A. Aquarium sign in the left atrium. Cardiology. 2007;107(4):411.
68. Belcher E, Lawson MH, Nicholson AG, Davison A, Goldstraw P. Congenital cystic adenomatoid malformation presenting as in-flight systemic air embolisation. The European respiratory journal. 2007;30(4):801-4.
69. Ghatge SB, Bhatgadde VL, Nagar AM, Raut AA, Narlawar RS. Paradoxical cerebral arterial gas embolism: Computed tomography findings. Australas Radiol. 2007;51 Suppl:B210-3.
70. Johnston SL, Halabi S, Cohoon K, Alex C, Hutchens K, Leya F. Stroke and myocardial infarction as late complications of lung transplantation. The Journal of heart and lung transplantation : the official publication of the International Society for Heart Transplantation. 2007;26(10):1065-8.
71. Khazei A, Harrison D, Abu-Laban RB, Mitra A. Potential missed cerebral arterial gas embolism in patients with in-hospital ischaemic stroke. Diving and Hyperbaric Medicine. 2007;37(2):58-64.
72. Krishnamoorthy T, Gupta AK, Rajan JE, Thomas B. Stroke from delayed embolization of polymerized glue following percutaneous direct injection of a carotid body tumor. Korean J Radiol. 2007;8(3):249-53.
73. Laguillo-Sala G, Cañete-Abajo N, Castaño-Duque CH, Guardia-Mas E, De Juan-Delago M, Ruscalleda-Nadal J. Cerebral gas embolism secondary to withdrawal of a central venous line. Revista de Neurologia. 2007;44(2):92-4.
74. Barak M, Kabha M, Norman D, Soudry M, Kats Y, Milo S. Cerebral microemboli during hip fracture fixation: a prospective study. Anesth Analg. 2008;107(1):221-5.
75. Breivik KL, Nielsen EW. [A 69-year-old man with temporary left-sided hemiparesis]. Tidsskrift for den Norske laegeforening : tidsskrift for praktisk medicin, ny raekke. 2008;128(22):2585-6.
76. Buompadre MC, Arroyo HA. Accidental cerebral venous gas embolism in a young patient with congenital heart disease. Journal of child neurology. 2008;23(1):121-3.
77. Bush RG, Derrick M, Manjoney D. Major neurological events following foam sclerotherapy. Phlebology. 2008;23(4):189-92.
78. Erasmus DB, Alvarez F, Keller CA. Fatal arterial gas embolism in an adult 1 year after bilateral sequential lung transplantation. The Journal of heart and lung transplantation : the official publication of the International Society for Heart Transplantation. 2008;27(6):692-4.
79. Hirasawa S, Hirasawa H, Taketomi-Takahashi A, Morita H, Tsushima Y, Amanuma M, et al. Air embolism detected during computed tomography fluoroscopically guided transthoracic needle biopsy. Cardiovasc Intervent Radiol. 2008;31(1):219-21.
80. Hsi DH, Thompson TN, Fruchter A, Collins MS, Lieberg OU, Boepple H. Simultaneous coronary and cerebral air embolism after CT-guided core needle biopsy of the lung. Texas Heart Institute journal / from the Texas Heart Institute of St Luke's Episcopal Hospital, Texas Children's Hospital. 2008;35(4):472-4.
81. Kau T, Rabitsch E, Celedin S, Habernig SM, Weber JR, Hausegger KA. When coughing can cause stroke--a case-based update on cerebral air embolism complicating biopsy of the lung. Cardiovasc Intervent Radiol. 2008;31(5):848-53.
82. Lau G. Fatal cerebral infarction complicating therapeutic embolisation of a facial cavernous haemangioma: a case report. Medicine, science, and the law. 2008;48(3):256-60.
83. Scruggs JE, Joffe A, Wood KE. Paradoxical air embolism successfully treated with hyperbaric oxygen. J Intensive Care Med. 2008;23(3):204-9.
84. Alexandrov AV, Fulton J, MacGregor G, Zhao L, Moehring M, Alexandrov AW. Spectral and power M-mode Doppler (PMD) signatures of symptomatic brain air embolism. Cerebrovascular Diseases. 2009;27:35.
85. Argüelles García B, García Blanco A, Meilán Martínez A, Calvo Blanco J. Cerebral artery air embolism secondary to endoscopic retrograde cholangiopancreatography. Gastroenterologia y Hepatologia. 2009;32(9):614-7.
86. Brockmeyer J, Simon T, Seery J, Johnson E, Armstrong P. Cerebral air embolism following removal of central venous catheter. Mil Med. 2009;174(8):878-81.
87. Cipriani NA, Hong C, Rosenblum J, Pytel P. Air embolism with pneumocephalus. Arch Neurol. 2009;66(9):1172-3.
88. Edwardson M, Wurth D, Lacy JM, Fink J, Becker K. Cerebral air embolism resulting in fatal stroke in an airplane passenger with a pulmonary bronchogenic cyst. Neurocrit Care. 2009;10(2):218-21.
89. Jeannin A, Saignac P, Palussière J, Gékière JP, Descat E, Lakdja F. Massive systemic air embolism during percutaneous radiofrequency ablation of a primary lung tumor. Anesthesia and Analgesia. 2009;109(2):484-6.
90. Kavalieros P, Reyneke E, Sturm J, Crimmins D. Air emboli causing acute ischaemic stroke after medical procedures. International Journal of Stroke. 2009;4:29-30.
91. Leslie-Mazwi TM, Avery LL, Sims JR. Intra-arterial air thrombogenesis after cerebral air embolism complicating lower extremity sclerotherapy. Neurocrit Care. 2009;11(2):247-50.
92. Mehta RI, Mehta RI, Fishbein MC, Solis OE, Jahan R, Salamon N, et al. Intravascular polymer material after coil embolization of a giant cerebral aneurysm. Human Pathology. 2009;40(12):1803-7.
93. Pellisé A, Ustrell X, Viñas J, Ruiz V, Guedea A, Sempere T, et al. Cerebral air embolism as a cause of stroke after removal of a central venous catheter. Cerebrovascular Diseases. 2009;27:132.
94. Seeburger J, Borger MA, Merk DR, Doll S, Bittner HB, Mohr FW. Massive cerebral air embolism after bronchoscopy and central line manipulation. Asian cardiovascular & thoracic annals. 2009;17(1):67-9.
95. Skjelland M, Krohg-Sorensen K, Tennoe B, Bakke SJ, Brucher R, Russell D. Cerebral microemboli and brain injury during carotid artery endarterectomy and stenting. Stroke. 2009;40(1):230-4.
96. Um SJ, Lee SK, Doo KY, Son C, Ki NK, Lee KN, et al. Four cases of a cerebral air embolism complicating a percutaneous transthoracic needle biopsy. Korean Journal of Radiology. 2009;10(1):81-4.
97. Wheen LC, Williams MP. Post-mortems in recreational scuba diver deaths: the utility of radiology. Journal of forensic and legal medicine. 2009;16(5):273-6.
98. Williams TL, Parikh DR, Hopkin JR, Lukovits TG, Kono AT, Mamourian AC, et al. Teaching NeuroImages: cerebral air embolism secondary to atrial-esophageal fistula. Neurology. 2009;72(12):e54-5.
99. Bou-Assaly W, Pernicano P, Hoeffner E. Systemic air embolism after transthoracic lung biopsy: A case report and review of literature. World journal of radiology. 2010;2(5):193-6.
100. Chemmanam T, Ghia D, Bladin C. Varicose vein sclerotherapy-an uncommon cause of stroke. International Journal of Stroke. 2010;5:41-2.
101. Delaney MC, Bowe CT, Higgins GL. Acute stroke from air embolism after leg sclerotherapy. The western journal of emergency medicine. 2010;11(4):397.
102. Galion A, Do AH, Chang GY. Lateralized infarction in cerebral air embolism due to patient positioning. Journal of clinical neuroscience : official journal of the Neurosurgical Society of Australasia. 2010;17(7):943-4.
103. Guillard E, Nancy B, Floch H, Henckes A, Cochard G, Arvieux J, et al. Intracerebral hemorrhage related to systemic gas embolism during hysteroscopy. Undersea Hyperb Med. 2010;37(2):89-93.
104. Huang Y, Sun W, Li F, Sun W. Azygos vein to pulmonary vein fistula is a pathway for cerebral embolism. Chest. 2010;138(3):726-9.
105. Hussain W, Salmon P, Gleeson C, Mortimer N. Collapse during scalp tumour extirpation: A cautionary tale for the Mohs micrographic surgeon. British Journal of Dermatology. 2010;163:104.
106. Jung S, Wiest R, Frigerio S, Mattle HP, Hess CW. Cerebral air embolism caused by a bronchogenic cyst. Pract Neurol. 2010;10(3):164-6.
107. Kiribayashi M, Nakasone M, Moriyama N, Mochida S, Yamasaki K, Minami Y, et al. [Multiple cerebral infarction by air embolism associated with remarkable low BIS value during lung segmentectomy with video assisted thoracic surgery (VATS) technique: a case report]. Masui The Japanese journal of anesthesiology. 2010;59(4):480-3.
108. Kwon JH, Shin YE. Cerebral air embolism after haemodialysis in a patient with atrial septal defect. Journal of Neurology. 2010;257:S159.
109. Lee JH, Kwon TD, Kim HJ, Kang B, Koo BN. Multiple cerebral infarction and paradoxical air embolism during hepatectomy using the Cavitron Ultrasonic Surgical Aspirator -A case report. Korean J Anesthesiol. 2010;59 Suppl:S133-6.
110. Mehta RI, Mehta RI, Solis OE, Jahan R, Salamon N, Tobis JM, et al. Hydrophilic polymer emboli: an under-recognized iatrogenic cause of ischemia and infarct. Modern pathology : an official journal of the United States and Canadian Academy of Pathology, Inc. 2010;23(7):921-30.
111. Miyamoto M, Inoue T, Nagata M, Yukawa H, Ogura M, Fujisawa T, et al. [A case report: multiple air embolism after the laryngopharyngoesophagectomy occurred by the cervical infection from postoperative fistula]. Nihon Jibiinkoka Gakkai kaiho. 2010;113(1):20-5.
112. Müller MCA, Lagarde SM, Germans MR, Juffermans NP. Cerebral air embolism after arthrography of the ankle. Medical Science Monitor. 2010;16(7):92-4.
113. Salameh J. A 62-year-old woman with cerebral artery air embolism during commercial air travel. Neurologist. 2010;16(2):136-7.
114. Seo SY, Lee DH, Hwang S, Kim SC, Hong KS, Cho JY, et al. Prominent inflammation and vasogenic edema in cerebral arterial air embolism: Serial MRI findings. International Journal of Stroke. 2010;5:184.
115. van Boxel GI, Hommers CE, Dash I, Goodman AJ, Green J, Orme RM. Myocardial and cerebral infarction due to massive air embolism following endoscopic retrograde cholangiopancreatography (ERCP). Endoscopy. 2010;42 Suppl 2:E80-1.
116. Zmistowski B, Austin L, Ciccotti M, Ricchetti E, Williams Jr G. Fatal venous air embolism during shoulder arthroscopy: A case report. Journal of Bone and Joint Surgery - Series A. 2010;92(11):2125-7.
117. Billinger M, Zbinden R, Mordasini R, Windecker S, Schwerzmann M, Meier B, et al. Patent foramen ovale closure in recreational divers: effect on decompression illness and ischaemic brain lesions during long-term follow-up. Heart. 2011;97(23):1932-7.
118. Cereda C, Staedler C, Moschovitis G, Caronni F, Bassetti CL, Azzola A. 'Bubbles in the brain': Systemic air embolism syndrome from an atrial-oesophageal fistula. Emergency Medicine Journal. 2011;28(5):455.
119. Clark DK, Plaizier E. Devastating cerebral air embolism after central line removal. The Journal of neuroscience nursing : journal of the American Association of Neuroscience Nurses. 2011;43(4):193-6; quiz 7-8.
120. French KF, Garcia C, Wold JJ, Hoesch RE, Ledyard HK. Cerebral air emboli with atrial-esophageal fistula following atrial fibrillation ablation: a case report and review. The Neurohospitalist. 2011;1(3):128-32.
121. Higashino T, Noma S, Nishimoto Y, Endo J, Taguchi Y, Shindo T. Cerebral air embolism as a complication of computed tomography-guided marking of the lung: Depiction of air inflow route from a pulmonary vein to the left atrium. Journal of Thoracic Imaging. 2011;26(1):W26-W9.
122. Jacobs M, Macedo FJ. Central poststroke pain syndrome secondary to embolization of shrapnel. Successful treatment with lidocaine infusion and oral mexiletine. A case report. PM and R. 2011;3(10):S284.
123. Kanth P, Fang J. Cerebral air embolism: An uncommon complication of a bleeding atrioesophageal fistula. American Journal of Gastroenterology. 2011;106:S374.
124. Karnatovskaia L. Stress-induced cardiomyopathy due to systemic air embolism-induced cerebrovascular accident. Critical Care Medicine. 2011;39:264.
125. Leong JCY, Johnston NR. Visual loss following sclerotherapy for varicose veins. BMJ Case Reports. 2011.
126. Lippmann J, Fock A, Arulanandam S. Cerebral arterial gas embolism with delayed treatment and a fatal outcome in a 14-year-old diver. Diving and Hyperbaric Medicine. 2011;41(1):31-4.
127. Ma RWL, Pilotelle A, Paraskevas P, Parsi K. Three cases of stroke following peripheral venous interventions. Phlebology. 2011;26(7):280-4.
128. Matte GS, Kussman BD, Wagner JW, Boyle SL, Howe RJ, Pigula FA, et al. Massive air embolism in a fontan patient. Journal of Extra-Corporeal Technology. 2011;43(2):79-83.
129. Moro PJ, Coulange M, Brissy O, Cuisset T, Quilici J, Mouret JP, et al. Acute coronary syndrome and cerebral arterial gas embolism in a scuba diver. Journal of Cardiology Cases. 2011;3(1):e22-e5.
130. Nascimbene A, Angelini P. Superior vena cava thrombosis and paradoxical embolic stroke due to collateral drainage from the brachiocephalic vein to the left atrium. Texas Heart Institute journal / from the Texas Heart Institute of St Luke's Episcopal Hospital, Texas Children's Hospital. 2011;38(2):170-3.
131. Regan JD, Gibson KD, Rush JE, Shortell CK, Hirsch SA, Wright DDI. Clinical significance of cerebrovascular gas emboli during polidocanol endovenous ultra-low nitrogen microfoam ablation and correlation with magnetic resonance imaging in patients with right-to-left shunt. Journal of Vascular Surgery. 2011;53(1):131-7.
132. Sharma T, Lall A, Panigrahi B. Case report-recognizing air embolism post hysteroscopic surgery. Respirology. 2011;16:267-8.
133. Tunel HA, Gulcan O. Succesfully treated Cerebral air embolism with hyperbaric oxygen therapy after coronary artery bypass grafting. Heart Surgery Forum. 2011;14:S77-S8.
134. Westwood AJ, Nguyen TN. Teaching NeuroImages: TIA from an air embolism. Neurology. 2011;77(21):e123.
135. Dedaj R, Preston IR. A case report: Acute stroke after echocardiogram with bubble study in a patient with patent foramen ovale and chronic thromboembolic pulmonary hypertension. American Journal of Respiratory and Critical Care Medicine. 2012;185.
136. Errico M, Singh A, Shade D, Zikos A, Belden W, Moraca R. Cerebral air embolism and atrio-esophageal fistula. Neurocritical Care. 2012;17:S184.
137. Jones D, Galvez-Jimenez N. An unusual cause of left hemiplegia in a renal transplant patient. Neurology. 2012;78(1).
138. Karnatovskaia LV, Lee AS, Dababneh H, Lin A, Festic E. Stress-induced cardiomyopathy complicating a stroke caused by an air embolism. Journal of bronchology & interventional pulmonology. 2012;19(3):224-7.
139. Kesieme E, Feldmann M, Welcker K, Linder A, Prisadov G. Cerebral infarct complicating traumatic pneumatocele: a rare sequela following blunt chest trauma. The Thoracic and cardiovascular surgeon. 2012;60 Suppl 2:e16-8.
140. Leschka SC, Schumacher M. [Severe, reversible cerebral ischaemia following a diving accident]. Deutsche medizinische Wochenschrift (1946). 2012;137(9):425-8.
141. Martins J, Casimiro C, Tomás J, Mendonc¸a N, Marnoto D. Cerebral ischemia: Potential complication of gas embolism after pulmonar biopsy? Neuroradiology. 2012;54(1):91-2.
142. Miyamoto S, Mashimo Y, Horimatsu T, Ezoe Y, Morita S, Muto M, et al. Cerebral air embolism caused by chemoradiotherapy for esophageal cancer. Journal of Clinical Oncology. 2012;30(25):e237-e8.
143. Oyama N, Sakaguchi M, Kitagawa K. Air tract in the thrombus: paradoxical cerebral air embolism through a residual catheter track. Journal of stroke and cerebrovascular diseases : the official journal of National Stroke Association. 2012;21(8):905.e11-3.
144. Parsi K. Paradoxical embolism, stroke and sclerotherapy. Phlebology. 2012;27(4):147-67.
145. Suzuki K, Ueda M, Abe A, Nishiyama Y, Okubo S, Katsura K, et al. Paradoxical cerebral air embolism occurred with postural change during rehabilitation, in a patient with ipsilateral internal carotid artery occlusion. Internal Medicine. 2012;51(9):1107-9.
146. Tanaka R, Shimada Y, Shimura H, Oizumi H, Hattori N, Tanaka S. Predominant vasogenic edema in a patient with fatal cerebral air embolism. Journal of Stroke and Cerebrovascular Diseases. 2012;21(6):509-11.
147. Taylor R, Benton C, Schneider A. Cerebral air embolism with upper endoscopy: Two patients, three events. Neurology. 2012;78(1).
148. Adatia S, Nambiar V, Kapadia R, Abuzinath A, Apel S, Alqarni M, et al. Acute ischemic stroke caused by paradoxical air embolism following injection sclerotherapy for varicose veins. Neurol India. 2013;61(4):431-3.
149. Akins P, Amar AP, Pakbaz S, Fields J. Peri-procedural complications of endovascular treatment for acute stroke in swift trial. Stroke. 2013;44(2).
150. Amar AP, Akins P, Fields J, Pakbaz S. Peri-procedural complications of endovascular treatment for acute stroke in swift trial. Journal of Neurosurgery. 2013;119(2):A536-A7.
151. Aryana A, Arthur A, Nieves MA, O'Neill PG, D'Avila A. Fatal manifestation of an atrio-esophageal fistula following a surgical 'mini-maze' procedure for treatment of atrial fibrillation. Heart Rhythm. 2013;10(5):S474.
152. Baban CK, Murphy M, Hennessy T, O'Hanlon D. Fatal cerebral air embolism following endoscopic evaluation of rectal stump. BMJ Case Reports. 2013.
153. Chavalitdhamrong D, Draganov PV. Acute stroke due to air embolism complicating ERCP. Endoscopy. 2013;45 Suppl 2 UCTN:E177-8.
154. Cortegiani A, Foresta G, Strano G, Strano MT, Montalto F, Garbo D, et al. An atypical case of Taravana syndrome in a breath-hold underwater fishing champion: A case report. Case Reports in Medicine. 2013;2013.
155. Dalal PK, Varma D. Hemiparesis and aphasia secondary to air embolism from a CT-guided lung biopsy-under-recognized, overly important. American Journal of Respiratory and Critical Care Medicine. 2013;187.
156. Dutta T, Srour D, Sheth K. Basilar artery air embolus complicating vein foam sclerotherapy. Neurology. 2013;80(1).
157. Fekih Hassen M, Ayed Dalla S, Ayed S, Tilouche N, Ben Sik Ali H, Gharbi R, et al. Delayed treatment of iatrogenic cerebral air embolism with hyperbaric oxygen. Trends in Anaesthesia and Critical Care. 2013;3(5):289-90.
158. Lai D, Jovin TG, Jadhav AP. Cortical vein air emboli with gyriform infarcts. JAMA Neurology. 2013;70(7):939-40.
159. Mirza S, Pope S. Multifocal embolic stroke in the setting of an open central venous catheter and an atrial septal aneurysm. American Journal of Respiratory and Critical Care Medicine. 2013;187.
160. Nakagawa EM, Freeman M, Eugeniu M. Rare case of ischemic stroke caused by air emboli from hydrogen peroxide ingestion. Journal of Neuroimaging. 2013;23(2):273.
161. Sebat CM, Albertson TE, Morrissey BM. Cerebral gas embolism in a case of Influenza A-associated acute respiratory distress syndrome treated with high-frequency oscillatory ventilation. Ann Thorac Med. 2013;8(2):124-6.
162. Sheth S, Paul R, Durrant J. Air in the brain: An unexpected complication of a misplaced peripheral intravenous line. Neurology. 2013;80(1).
163. Shi L, Zhang R, Wang Z, Zhou P. Delayed cerebral air embolism complicating percutaneous needle biopsy of the lung. The American journal of the medical sciences. 2013;345(6):501-3.
164. Smith K, Alawi A, Kalia J, Hadid M, Mikolajczak P, Feen E. Diffuse multiple air emboli secondary to atrial-esophageal fistula: A complication of atrial fibrillation ablation therapy. Neurocritical Care. 2013;19(1):S298.
165. Tsetsou S, Eeckhout E, Qanadli SD, Lachenal Y, Vingerhoets F, Michel P. Non-accidental arterial cerebral air embolism: A 10-year stroke center experience. Cerebrovascular Diseases. 2013;35:644.
166. Yeung JTH, Ma JKF, Mak YF, Lam VSC. Fatal cerebral air embolism related to an air flight. Hong Kong Medical Journal. 2013;19(4):352-3.
167. Yin JH, Chuang YJ, Hu HH. Pneumocephalus associated with massive cerebral air embolism. Acta Neurol Taiwan. 2013;22(2):93-4.
168. Ahmed I, Myers T, Kim J. An unusual cause of altered mental status: Recurrent cerebral air embolism from atrial-esophageal fistula. Neurology. 2014;82(10).
169. Akins PT, Amar AP, Pakbaz RS, Fields JD. Complications of endovascular treatment for acute stroke in the SWIFT trial with solitaire and Merci devices. AJNR Am J Neuroradiol. 2014;35(3):524-8.
170. Bowles PF, Lear C, Maccario M, Kong R. Paradoxical air embolism and neurological insult during removal of a pulmonary artery catheter introducer. BMJ Case Rep. 2014;2014.
171. Cross D, Axelband J. Cerebral air embolism, stroke and status epilepticus following PICC insertion. Critical Care Medicine. 2014;42(12):A1647.
172. Dong H, Grimes B, Oh S, Susanto I. Airways and airheads: A case of bronchoscopy-induced cerebral arterial gas embolism. Chest. 2014;146(4).
173. Gempp E, Louge P, Soulier B, Alla P. Cerebellar infarction presenting as inner ear decompression sickness following scuba diving: a case report. Eur Ann Otorhinolaryngol Head Neck Dis. 2014;131(5):313-5.
174. Hegland DL, Canales BK, Katz RI. Quadriparesis from air emboli during percutaneous nephrolithotomy. Journal of Clinical Anesthesia. 2014;26(4):318-20.
175. Huang C, Pik J. Tension pneumocephalus and oxygen emboli from hydrogen peroxide irrigation. Journal of Clinical Neuroscience. 2014;21(2):323-5.
176. Khan M, Hassan O, Fine M. Unusual complications related to endoscopic retrograde cholangiopancreatography with a focus on air embolism. American Journal of Gastroenterology. 2014;109:S450.
177. Lin C, Barrio GA, Hurwitz LM, Kranz PG. Cerebral air embolism from angioinvasive cavitary aspergillosis. Case reports in neurological medicine. 2014;2014:406106.
178. Moyer L. Progressive embolic infarction secondary to air emboli from atrioesophageal fistula status post left atrial ablation for atrial fibrillation: A case report. Chest. 2014;146(4).
179. Patankar PS, Joshi SS, Choudhari KA. Air-embolism and cerebral ischaemia following epidural hydrogen peroxide irrigation in a closed lumbar cavity. Br J Neurosurg. 2014;28(4):556-8.
180. Shaikh N, Ramanathan RS, Dike N, Malhotra K, Rana S. Embolic stroke post ablation due to atrioesophageal fistula. Journal of Neuroimaging. 2014;24(3):319.
181. Shin KM, Lim JK, Kim CH. Delayed presentation of cerebellar and spinal cord infarction as a complication of computed tomography-guided transthoracic lung biopsy: A case report. Journal of Medical Case Reports. 2014;8(1).
182. Suri V, Gupta R, Sharma G, Suri K. An unusual cause of ischemic stroke - Cerebral air embolism. Ann Indian Acad Neurol. 2014;17(1):89-91.
183. Thapa J, Dalal PK, Varma D. Hyperbaric oxygen therapy for cerebral artery air embolism-a rare complication of percutaneous needle lung biopsy. American Journal of Respiratory and Critical Care Medicine. 2014;189.
184. Wiesen J, Wiesen A, Tsuang W. All the bubbles “are-gone”: An unusual cause of stroke post argon plasma coagulation in a patient with hereditary hemorrhagic telangiectasia (HHT). Chest. 2014;146(4).
185. Yesilaras M, Atilla OD, Aksay E, Kilic TY. Retrograde cerebral air embolism. American Journal of Emergency Medicine. 2014;32(12):1562.e1-.e2.
186. Aghaebrahim A, Giurgiutiu DV, Jankowitz BT, Jovin T, Jadhav AP. Ischemic stroke after pellet embolization. Neurology. 2015;84(23):2383.
187. Arnott C, Kelly K, Wolfers D, Cranney G, Giles R. Paradoxical cardiac and cerebral arterial gas embolus during percutaneous lead extraction in a patient with a patent foramen ovale. Heart, lung & circulation. 2015;24(1):e14-7.
188. Delgado HM, Barbosa R, Inacio NMO, Cavaco D, Calado SL. Stroke after radiofrequency catheter ablation for atrial fibrillation: The role of atrio-esophageal fistula. European Journal of Neurology. 2015;22:799.
189. Ferdous J, Tantikittichaikul S, Hasan R, Eldokla A, Kim J. Symptomatic cerebral air embolism after central venous catheter removal. Neurology. 2015;84.
190. Gillette M, Putnam PJ. Air on the mind, a case of nontraumatic pneumocephalus. Journal of General Internal Medicine. 2015;30:S354.
191. Gudmundsdottir JF, Geirsson A, Hannesson P, Gudbjartsson T. Major ischaemic stroke caused by an air embolism from a ruptured giant pulmonary bulla. BMJ Case Rep. 2015;2015.
192. Hanke I, Kodýtková A, Penka I. Air embolism of the brain - A case report. Ceska a Slovenska Neurologie a Neurochirurgie. 2015;78(3):359-62.
193. Hung WH, Chang CC, Ho SY, Liao CY, Wang BY. Systemic air embolism causing acute stroke and myocardial infarction after percutaneous transthoracic lung biopsy-a case report. J Cardiothorac Surg. 2015;10:121.
194. Kaichi Y, Kakeda S, Korogi Y, Nezu T, Aoki S, Matsumoto M, et al. Changes over Time in Intracranial Air in Patients with Cerebral Air Embolism: Radiological Study in Two Cases. Case reports in neurological medicine. 2015;2015:491017.
195. Koa-Wing M, Jamil-Copley S, Ariff B, Kojodjojo P, Lim PB, Whinnett Z, et al. Haemorrhagic cerebral air embolism from an atrio-oesophageal fistula following atrial fibrillation ablation. Perfusion (United Kingdom). 2015;30(6):484-6.
196. Mansoor MS, Usmani A. Cerebral air embolism complicating esophageal dilation. American Journal of Gastroenterology. 2015;110:S277-S8.
197. Jens W, Lee A, Ibrahimi M. Imaging of a fatal air embolism from ERCP. Neurology. 2016;86(16).
198. Kale P, Javed B, Pednekar N, Sahni R, Resor L, Tenner M, et al. Stroke due to air embolism related to laser ablation of accessory vein. Neurology. 2016;86(16).
199. Lorentzen AO, Nome T, Bakke SJ, Scheie D, Stenset V, Aamodt AH. Cerebral foreign body reaction after carotid aneurysm stenting. Interventional neuroradiology : journal of peritherapeutic neuroradiology, surgical procedures and related neurosciences. 2016;22(1):53-7.
200. Parikh D, Leyon JJ, Chavda S. Gas Embolic Stroke Secondary to Bowel Infarction. Journal of stroke and cerebrovascular diseases : the official journal of National Stroke Association. 2016;25(1):e1-3.
201. Park S, Ahn JY, Ahn YE, Jeon SB, Lee SS, Jung HY, et al. Two Cases of Cerebral Air Embolism That Occurred during Esophageal Ballooning and Endoscopic Retrograde Cholangiopancreatography. Clinical endoscopy. 2016;49(2):191-6.
202. Pinho J, Amorim JM, Araujo JM, Vilaca H, Ribeiro M, Pereira J, et al. Cerebral gas embolism associated with central venous catheter: Systematic review. J Neurol Sci. 2016;362:160-4.
203. Rehwald R, Loizides A, Wiedermann FJ, Grams AE, Djurdjevic T, Glodny B. Systemic air embolism causing acute stroke and myocardial infarction after percutaneous transthoracic lung biopsy - a case report. J Cardiothorac Surg. 2016;11(1):80.
204. 1: Kanchustambham V, Reddy M, Saladi S, Patolia S. Cerebral Air Embolism as

Possible Cause of Stroke During Therapeutic Endobronchial Application of Argon

Plasma Coagulation. Cureus. 2017 May 17;9(5):e1255.

1. Kawahara T, Hagiwara M, Takahashi H, Tanaka M, Imai K, Sawada J, Kunisawa T,

Furukawa H. Cerebral Infarction by Paradoxical Gas Embolism During Laparoscopic

Liver Resection with Injury of the Hepatic Vessels in a Patient without a

Right-to-Left Systemic Shunt. Am J Case Rep. 2017 Jun 20;18:687-691.

1. Farshchi Zarabi S, Parotto M, Katznelson R, Downar J. Massive Ischemic Stroke Due to Pulmonary Barotrauma and Cerebral Artery Air Embolism During Commercial Air Travel. Am J Case Rep. 2017 Jun 13;18:660-664.
2. Voigt P, Schob S, Gottschling S, Kahn T, Surov A. Systemic air embolism after

endoscopy without vessel injury - A summary of reported cases. J Neurol Sci. 2017

May 15;376:93-96.

1. Ohuchi M, Inoue S, Ozaki Y, Ueda K. Systemic air embolism during pleural

lavage for empyema. Gen Thorac Cardiovasc Surg. 2016 Dec 16. [Epub ahead of

print] PubMed PMID: 27987101.

1. Shah J, Jiwa N, Mamdani N, Hill D. Venous and arterial air embolism: a rare

phenomenon with fatal consequences. BMJ Case Rep. 2016 Dec 5;2016.

1. Zwirner J, Bayer R, Hädrich C, Bollmann A, Klein N, Dreßler J, Ondruschka B.

Pulmonary artery perforation and coronary air embolism-two fatal outcomes in

percutaneous left atrial appendage occlusion. Int J Legal Med. 2017

Jan;131(1):191-197.

1. Rohlffs F, Tsilimparis N, Saleptsis V, Diener H, Debus ES, Kölbel T. Air

Embolism During TEVAR. J Endovasc Ther. 2017 Feb;24(1):84-88.

1. Roquero LP, Camelo-Piragua S, Schmidt C. Cerebral Air Embolism: A Clinical,

Radiologic and Histopathologic Correlation. Am J Forensic Med Pathol. 2016

Dec;37(4):241-244.

1. Tabata H, Kitaguchi H, Terajima Y, Shindo K. Cerebral Air Embolism with

Pneumomediastinum Resulting from Emesis: A Case Report. J Stroke Cerebrovasc Dis.

2016 Oct;25(10):e178-80.

1. Belton PJ, Nanda A, Alqadri SL, Khakh GS, Chandrasekaran PN, Newey C,

Humphries WE. Paradoxical cerebral air embolism causing large vessel occlusion

treated with endovascular aspiration. J Neurointerv Surg. 2017 Apr;9(4):e10.

1. Tetzlaff K, Schöppenthau H, Schipke JD. Risk of Neurological Insult in

Competitive Deep Breath-Hold Diving. Int J Sports Physiol Perform. 2017

Feb;12(2):268-271. doi: 10.1123/ijspp.2016-0042. Epub 2016 Aug 24.

1. Rehwald R, Loizides A, Wiedermann FJ, Grams AE, Djurdjevic T, Glodny B.

Systemic air embolism causing acute stroke and myocardial infarction after

percutaneous transthoracic lung biopsy - a case report. J Cardiothorac Surg. 2016

May 6;11(1):80. doi: 10.1186/s13019-016-0478-z. PubMed PMID: 27154545;

Search Criteria

Embase Search Criteria

('air embolism'/exp OR 'gas embolism'/exp OR 'foreign body'/exp) AND ('cerebrovascular accident'/exp OR 'brain infarction'/de OR 'brain ischemia'/exp)

((debris NEAR/3 embol*):ab,ti OR ('barotrauma'/exp OR 'diving'/exp) ) AND (('cerebrovascular accident'/exp OR 'brain infarction'/de OR 'brain ischemia'/exp) OR ((Cerebral OR brain) NEAR/3 (infarc* OR ischemi*)):ti,ab)

PUBMED Search Criteria

(Brain infarction*[tiab] OR brain stem infarction*[tiab] OR cerebral infarction*[tiab] OR stroke*[tiab] OR Cerebrovascular Accident*[tiab] OR brain ischemia*[tiab] OR "Brain Ischemia"[Mesh] OR "Stroke"[Mesh]) AND (Air embol*[tiab] OR gas embol*[tiab] OR foreign bod*[tiab] OR "Embolism, Air"[Mesh] OR "Foreign Bodies"[Mesh] OR debris[tiab] OR "Decompression Sickness"[Mesh] OR bends[tiab])

PRISMA Flow Diagram

Studies included in quantitative synthesis (meta-analysis)
(n = 216 )

Studies included in qualitative synthesis
(n = 216 )

Full-text articles excluded, with reasons
(n = 32 )

Full-text articles assessed for eligibility
(n = 248 )

Records excluded
(n = 1593 )

Records screened
(n = 297 )

Records after duplicates removed
(n = 1889 )

Additional records identified through other sources
(n = 1 )

## Identification

## Eligibility

## Included

## Screening

Records identified through database searching
(n = 2414 )
